# Supplementary material for: End Sequence Analysis Toolkit (ESAT) expands the extractable information from single-cell RNA-seq data
Source: Genome Res. 2016 Oct;26(10):1397–410. doi: 10.1101/gr.207902.116 (PMC5052061; doi:10.1101/gr.207902.116)
Supplement: Supplemental Material [file supp_gr.207902.116_Supp_Materials.tar.gz › ESAT-esat_v0.1/doc/html/functions_func_h.html]

ESAT: Class Members - Functions


ESAT


- Main Page
- Related Pages
- Packages
- Classes
- Files

- Class List
- Class Index
- Class Hierarchy
- Class Members

- All
- Functions
- Variables
- Enumerator

- a
- b
- c
- d
- e
- f
- g
- h
- i
- j
- k
- l
- m
- n
- o
- p
- q
- r
- s
- t
- u
- v
- w
- z


All Classes Namespaces Files Functions Variables Enumerator Pages

### - h -

- HairpinKmer()
  : broad.core.primer3.HairpinKmer
- hamming()
  : broad.core.math.Statistics
- hasAnnotationThatContains()
  : umms.core.annotation.AnnotationList< T extends Annotation >
- hasAnnotationThatOverlaps()
  : umms.core.annotation.AnnotationList< T extends Annotation >
- hasAnOverlappingGene()
  : umms.core.scripture.BuildScriptureCoordinateSpace
- hasBooleanFlag()
  : broad.core.parser.CommandLineParser
- hasCDS()
  : umms.core.annotation.Gene
- hasChromosome()
  : umms.core.coordinatesystem.CoordinateSpace
  , umms.core.coordinatesystem.GenomicSpace
  , umms.core.coordinatesystem.MaskedGenomicSpace
  , umms.core.coordinatesystem.TranscriptInGenomicSpace
  , umms.core.coordinatesystem.TranscriptomeSpace
- hasColumn()
  : broad.core.datastructures.MatrixWithHeaders
- hasDataForChromosome()
  : broad.pda.seq.segmentation.ContinuousDataAlignmentModel
- hasDoubleFlag()
  : broad.core.parser.CommandLineParser
- hasEntries()
  : umms.core.alignment.AlignmentPair
- hasFloatFlag()
  : broad.core.parser.CommandLineParser
- hashCode()
  : broad.core.annotation.BasicLightweightAnnotation
  , broad.core.datastructures.Pair< T1 >
  , broad.core.motif.SequenceMotif
  , broad.pda.seq.clip.SampleData
  , broad.pda.seq.graph.Path
  , broad.pda.seq.protection.SampleData
  , umms.core.alignment.FeatureSequenceAlignment.UnorderedGenePair
  , umms.core.alignment.FragmentAlignment
  , umms.core.alignment.PairedReadAlignment
  , umms.core.annotation.Gene
  , umms.core.model.score.CountScore
  , umms.core.readFilters.SplicedReadFilter
- hasIndel()
  : umms.core.alignment.AbstractPairedEndAlignment
  , umms.core.alignment.Alignment
  , umms.core.alignment.SingleEndAlignment
- hasIndex()
  : broad.pda.samtools.SAMPairedEndFileReader
  , umms.core.readers.PairedEndReader
- hasIntFlag()
  : broad.core.parser.CommandLineParser
- hasMore()
  : broad.core.math.CombinationGenerator
  , broad.pda.seq.fastq.FastqSplitterByBarcode
- hasNanostringProbeClasses()
  : broad.core.datastructures.MatrixWithHeaders
- hasNext()
  : broad.core.datastructures.IntervalTree< V >.FwdIterator
  , broad.core.datastructures.IntervalTree< V >.OverlapIterator
  , broad.core.datastructures.IntervalTree< V >.RevIterator
  , broad.core.datastructures.JCSCache.JCSNodeIterator
  , broad.core.datastructures.ReversibleIterator< E >
  , broad.core.sequence.WindowSlider
  , broad.pda.samtools.PairedEndReadIterator
  , broad.pda.samtools.WrappedIterator
  , broad.pda.seq.fastq.FastqParser
  , broad.pda.seq.segmentation.GenomeWithGaps
  , broad.pda.seq.segmentation.WrappedIGVIterator
  , umms.core.coordinatesystem.ShuffledIterator< T extends Annotation >
  , umms.core.general.CloseableFilterIterator< T >
  , umms.core.model.AlignmentModel.FilteredIterator
  , umms.core.model.AlignmentModel.NodeIterator
  , umms.core.model.AlignmentModel.WrapAlignmentCountIterator
  , umms.core.model.JCSAlignmentModel.FilteredIterator
  , umms.core.model.JCSAlignmentModel.NodeIterator
  , umms.core.model.JCSAlignmentModel.WrapAlignmentCountIterator
  , umms.core.model.score.WindowScoreIterator< T extends WindowScore >
- hasNextExon()
  : broad.pda.seq.segmentation.AlignmentDataModel
  , broad.pda.seq.segmentation.GenericAlignmentDataModel
- hasOrientation()
  : broad.core.sequence.SequenceRegion
  , umms.core.annotation.AbstractAnnotation
  , umms.core.annotation.Annotation
- hasOverlapping3PUTR()
  : broad.pda.gene.GeneWithIsoforms
- hasOverlapping5PUTR()
  : broad.pda.gene.GeneWithIsoforms
- hasRow()
  : broad.core.datastructures.MatrixWithHeaders
- hasStringFlag()
  : broad.core.parser.CommandLineParser
- hasSubClusters()
  : broad.pda.geneexpression.clustering.Cluster
- hasValue1()
  : broad.core.datastructures.Pair< T1 >
- hasValue2()
  : broad.core.datastructures.Pair< T1 >
- HierarchicalClustering()
  : broad.pda.geneexpression.clustering.HierarchicalClustering
- hochbergPvalueAdjust()
  : broad.core.math.Statistics
- hypergeometric()
  : broad.core.math.Statistics
- hypot()
  : broad.core.math.MathUtil
  , Jama.util.Maths


---

Generated on Fri Aug 15 2014 15:11:16 for ESAT by  

 1.8.7
